# Sampling trabecular meshwork using TrabEx+

**DOI:** 10.1186/s12886-021-01895-6

**Published:** 2021-03-19

**Authors:** Vipul Ramjiani, Hardeep-Singh Mudhar, Thomas Julian, Graham Auger

**Affiliations:** 1 Department of Ophthalmology, Sheffield Teaching Hospitals NHS Foundation Trust, Royal Hallamshire Hospital, Glossop Rd, England S10 2JF Sheffield, UK; 2 National Specialist Ophthalmic Pathology Service (NSOPS), Dept of Histopathology-E-Floor, Royal Hallamshire Hospital, Glossop Rd, England S10 2JF Sheffield, UK; 3 The University of Sheffield, Broomhall, England S10 2TG Sheffield, UK

**Keywords:** TrabEx+, Trabecular meshwork, Ab interno trabeculectomy, Histology, Tissue sampling

## Abstract

**Background:**

To report sampling of the trabecular meshwork using the TrabEx+ (MicroSurgical Technology, Redmond, Washington, USA) device in ab interno trabeculectomy. Specifically, this series focusses upon preservation of the trabecular meshwork architecture for assessment of glaucomatous features using common histopathological techniques.

**Patients:**

This series features six glaucomatous eyes undergoing TrabEx+ with or without cataract surgery. Three patients had primary open angle glaucoma and the remaining had pigment dispersion glaucoma, ocular hypertension or uveitic glaucoma. Four eyes had simultaneous cataract surgery.

**Methods:**

Trabecular meshwork was excised using the TrabEx+ device and retrieved using vitreoretinal forceps. The samples were then processed into formalin-fixed paraffin-embedded 4 micron tissue segments and stained with haematoxylin and eosin, periodic acid–Schiff and elastin Van Gieson. Collagen IV was labelled using immunohistochemistry for the purpose of identifying the basement membrane of trabecular beams.

**Results:**

Presence of trabecular meshwork was confirmed in five of the six samples taken. One of six samples consisted of blood only, but this was expected following early termination of the procedure due to patient restlessness. In the five positive cases trabecular beams with associated trabecular meshwork cells were identified on hematoxylin-eosin and periodic acid–Schiff staining. The beams retained their lamellar structure. The basement membrane underlying the trabecular cells was evident in three specimens, whilst two specimens were of insufficient size for collagen IV labelling.

**Conclusions:**

This case series illustrates that TrabEx+ can be utilised to successfully retrieve trabecular meshwork samples with sufficient architectural perseveration of the tissue to enable histopathological and laboratory analysis.

## Précis

In this case series, it has been demonstrated that the TrabEx + has the capacity to successfully retrieve architecturally preserved samples of trabecular meshwork during ab interno trabeculectomy.

## Background

TrabEx+ (MicroSurgical Technology, Redmond, Washington, USA) is a minimally invasive glaucoma surgery device, formally known as Goniotome + I/A (Neomedix USA). The design includes a serrated dual blade which simultaneously lifts and excises a strip of trabecular meshwork (TM). An ab interno approach is adopted though a temporal clear corneal incision. Up to 180 degrees of nasal TM is excised to facilitate aqueous outflow. The dual blade is supported by irrigation and aspiration (IA) ports which enables maintenance of a deep, stable and clear anterior chamber for better angle visualisation during surgery[1]. An example of another ab interno trabeculectomy device with IA support is the Trabectome (MicroSurgical Technology), however this device cannot be utilised to harvest TM due to its ablative function. The irrigation and aspiration support differentiates these two devices from other ab interno trabeculectomy instruments which require viscoelastic to enable gonioscopic view. Viscoelastic-dependent devices include standalone TrabEx (Microsurgical Technology) and Kahook Dual Blade (KDB) (New World Medical, Rancho Cucamonga, California, USA). All four procedures can be performed with or without cataract surgery. The outcomes of TrabEx + procedures are yet to be published; however our results (unpublished) show that they resemble the outcomes of Trabectome [2].

These four goniectomy devices were developed to address outflow resistance at the TM as a driver in primary open angle glaucoma (POAG). Although the pathogenesis of outflow resistance remains poorly understood, many changes in the structure of the TM have been documented in glaucomatous eyes. Pathological findings described in the literature include progressive TM cell loss, hyperpigmentation and extracellular matrix compositional change in relation to glycosaminoglycans and the elastic fibre network[3–9]. There is ongoing need for *in vivo* and *in vitro* studies of the outflow pathway in order to drive targeted advances in the medical and surgical treatment of glaucoma. The majority of histopathological studies of the TM and Schlemm’s canal (SC) are from cadaver specimens and ab externo trabeculectomy specimens. More recently, KDB has been shown to harvest TM with good yield[10]. TrabEx + includes a dual blade system similar to KDB and so also has the potential to harvest tissue, therefore enabling this device to retrieve free TM for histological analysis. The support of IA in the TrabEx + has the added benefit of improved angle visualisation during tissue excision when compared to the KDB, thus maximising degrees of TM excision and minimising inadvertent tissue damage[1]. An ophthalmic viscosurgical device (OVD) is used thereafter to maintain anterior chamber depth whilst TM is removed with forceps.

To the best of our knowledge, there is no publication addressing histological analysis of samples derived from TrabEx+. In this study, we describe a series of cases which underwent TrabEx + and the related histopathological findings. The primary outcome of this research was assessment of the preservation of the TM architecture after sampling. Secondary outcomes included the description of histopathological findings and a brief summary of the IOP and medication reduction after surgery.

## Methods

### Study design

This study was a non-interventional consecutive case series of six glaucomatous eyes undergoing TrabEx + with or without cataract surgery. All patients consented to involvement in the study. Surgeries were performed between June 2019 and September 2019. Tissue samples were collected from all eyes and sent for histopathological assessment to the National Specialist Ophthalmic Pathology Service (NSOPS) laboratory in Sheffield UK.

### Clinical assessment

Preoperative demographic information, diagnosis, past ocular surgery and previous laser trabeculoplasty history if applicable were collected. The Hodapp-Anderson-Parrish criteria for glaucoma severity were calculated for each eye accompanied by the Humphrey visual field mean deviation [11]. Eyes with ocular hypertension (OHT) had no severity grading. Intraocular pressures and the number of IOP lowering medications were collected before TrabEx + and at the latest follow up. Mean and standard deviations are documented for continuous variables.

### Surgical procedure

All surgeries were performed by one glaucoma surgeon, in one U.K. hospital trust. During surgery, the patients head is rotated 30 degrees away from the surgeon and the operating microscope is titled towards the surgeon. A temporal 1.8mm clear corneal incision is made. A goniolens is held in contact with the cornea with the aid of a viscoelastic coupling agent. The TrabEx + handpiece is inserted through the corneal wound and irrigation is engaged. The tip is advanced across the anterior chamber. The dual blades enter the nasal SC. TM is excised up to 90 degrees in a counter-clockwise direction. The dual blades are disengaged from SC, rotated 180 degrees, and reengaged with SC at the initial contact point. TM is excised up to 90 degrees in a clockwise direction. Two free ends of TM should be visible in the anterior chamber. The TrabEx + handpiece is withdrawn from the eye. Viscoelastic is injected into the AC. The free strips of TM are retrieved with vitreoretinal forceps. The TM tissue was placed into a formalin pot and sent for histology.

If eyes had simultaneous cataract surgery, this was performed prior to TrabEx+. Miochol was injected at the end of cataract surgery and the main wound was closed with one 10 − 0 nylon suture. A second 1.8mm wound anterior to the cataract wound was created for the TrabEx + handpiece and the procedure proceeds as previously described. Creating a second smaller wound for the TrabEx + handpiece minimises reflux from the AC leading to instability in AC depth compromising gonioscopic surgery.

### Histological preparation

The trabecular meshwork specimens were fixed overnight in 10 % buffered formalin, processed to paraffin blocks and 4 micron sections cut. The samples underwent routine staining with haematoxylin and eosin (H&E), periodic acid-Schiff (PAS), and elastin Von Gieson (EVG). Collagen IV was labelled in order to confirm the basement membrane of trabecular beams. Immunohistochemistry (IHC) for collagen IV (Clone CIV 22 Manufacturer Dako (Agilent) concentrate dilution 1:400) was completed as follows: Target Retrieval was carried out with EnVision FLEX TRS, Low pH (Dako Omnis) at 97 °C for 30 min incubation, followed by Primary Antibody incubation for 20 min. Then the endogenous enzyme block was carried out with EnVision FLEX Peroxidase blocking reagent (Dako Omnis) for 3 min, followed by labelled polymer, EnVision FLEX/HRP (Dako Omnis) for 20 min. Finally, substrate chromogen: EnVision FLEX Substrate Working Solution (Dako Omnis) was performed for 5 min before counterstaining with hematoxylin (Dako Omnis) for 3 min.

## Results

### Clinical context

The mean age was 69.8 ± 12.3 years. The mean time from diagnosis to surgery was 97.26 ± 104.54 months. Three patients had POAG and the remaining had pigment dispersion glaucoma (PDS), secondary uveitic glaucoma or OHT. Four eyes had simultaneous cataract surgery. Two eyes were pseudophakic at the time of TrabEx+. One eye had argon laser trabeculoplasty 24 years prior to TrabEx+ (Table 1). No other surgical procedures were performed on the eyes prior to TrabEx+. Pre-operative mean IOP and medications were 30.17mmHg (± 7.68mmHg) and 1.83 (± 1.72) respectively. No washout period was performed. Post-operative mean IOP and medications were 22.83mmHg (± 8.80mmHg) and 1.50 (± 1.76) respectively. The mean interval between operation and latest IOP and medication count follow up was 6.7 months (± 4.23). Thus, changes in mean IOP were − 7.33mmHg (p = 0.15), equivalent to a 24.31 % reduction; and medications were reduced by -0.33 (*p* = 0.75). Eye number 1 had no change in IOP and remained uncontrolled at 38mmHg. She was intolerant of any drops and was subsequently listed for trabeculectomy. Post trabeculectomy the eye remains controlled at 19mmHg off drops.

**Table 1:** Preoperative Clinical Features

Eye Number	Sex	Age (y)	Diagnosis	Combined Phacoemulsification	Laser Trabeculoplasty Type / Interval before TrabEx+ (y)	Diagnosis to Surgery Interval (m)	Glaucoma Severity^a^	HVF MD (dB)
1	F	40–50	OHT	-	N/A	72	n/a	-2.09
2	F	70–80	Uveitic	+	N/A	39	Early	-3.17
3	F	70–80	POAG	+	N/A	30	Early	-2.51
4	F	70–80	POAG	+	N/A	119	Early	-4.22
5	F	80–90	POAG	+	N/A	26	Moderate	-7.51
6	M	60–70	PDG	-	ALT / 24	298	Moderate	-7.68

*POAG *primary open angle glaucoma, *PDG *pigment dispersion glaucoma, *OHT *Ocular Hypertension, *ALT *argon laser trabeculoplasty, *HVF MD *Humphrey visual field mean deviation

^a^Determined using the Hodapp-Anderson-Parrish criteria

The process of harvesting tissue did not lead to complications but invariably added complexity and time to the procedure. This was due to blood reflux from the collector channels obscuring the angle view and patients becoming restless, presumably secondary to fluctuations in anterior chamber depth and extended surgical time.

### Histological findings

**Fig. 1:**
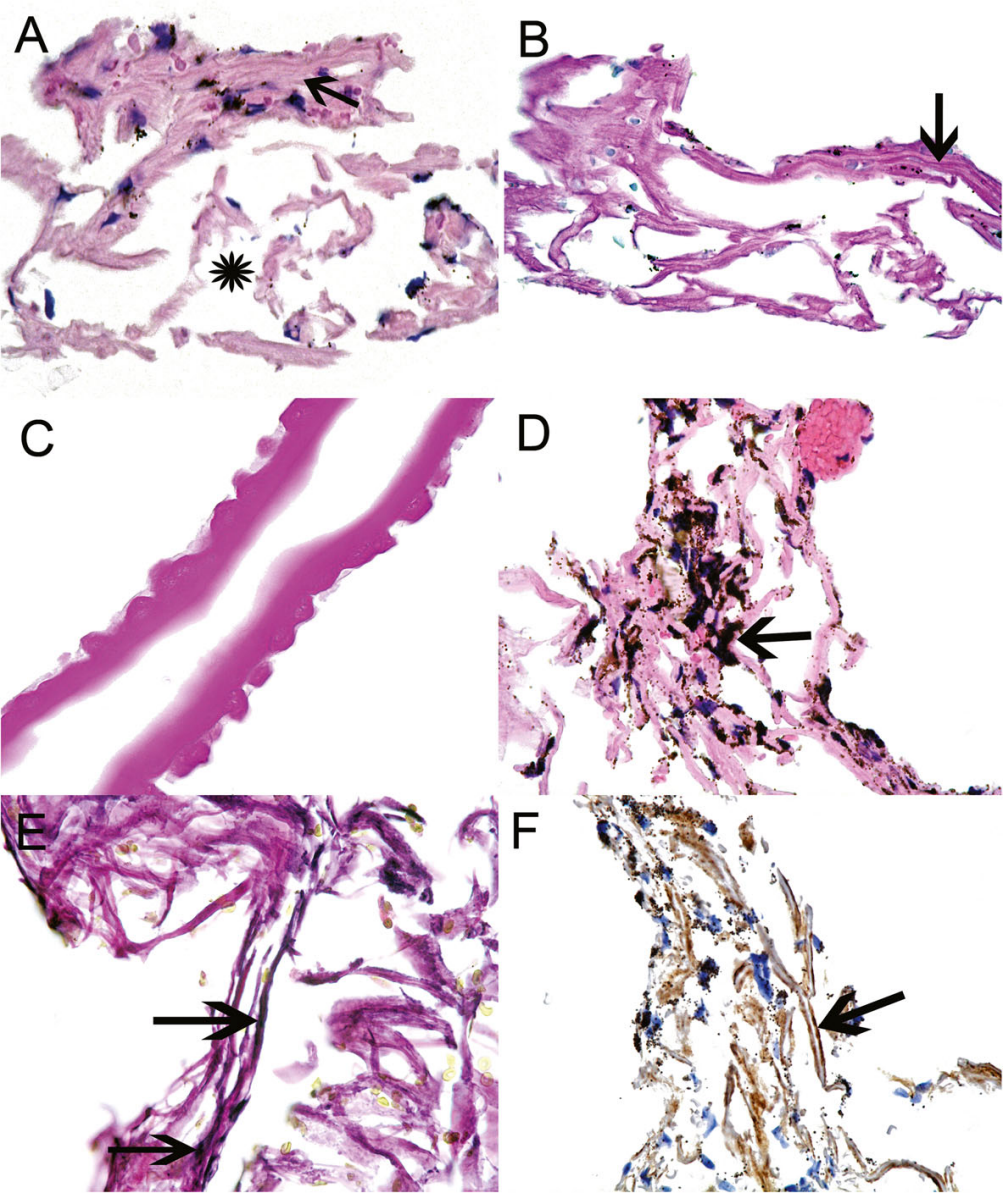
**a**-Haematoxylin and Eosin (H&E) stained section of trabecular meshwork. The arrow points to compressed trabecular beams. The asterisk indicates the spaces created by dispersion of the trabecular beams from surgical sampling. **b**-Periodic acid-Schiff (PAS) stain of the trabecular meshwork. The beams are stained pink. The arrow points to a layer of darker pink material on the trabecular beams indicating trabecular meshwork cell basement membrane. **c**-PAS stain showing the presence of Descemet’s membrane exhibiting Hassall-Henle excrescences. **d**-H&E stained trabecular meshwork from eye number 6 with pigment dispersion glaucoma. Note the dark brown pigment deposited on the trabecular beams (arrow). **e**-Elastin Von Gieson stain of trabecular meshwork showing patchy elastin presence (arrows point to black linear elastin). **f**-Collagen IV immunohistochemistry. The arrow points to tram track linear staining (brown-positive) over the beams.

Trabecular meshwork was confirmed on histology in five of the six specimens taken (Table 2). In the five positive cases trabecular beams with associated TM cells were identified on H&E and PAS staining (Fig. 1 a, b). The beams retained their lamellar structure. Some of the samples were distorted which was attributed to either instrumental handling in theatre or in the histopathology laboratory. The specimen from eye number 2 showed blood only and no evidence of trabecular meshwork. The histology outcome was predicted by the surgeon as tissue sampling was abandoned prematurely when the patient became restless. Three out of the five positive specimens demonstrated the presence of erythrocytes, an expected consequence of the surgical technique.

**Table 2:** Histology characteristics of trabecular meshwork samples derived from TrabEx+

Eye number	Structural Preservation of Trabecular Beams	Elastin with EVG staining	Collagen 4 present
1	Yes	No	n/a
2	Acellular	n/a	n/a
3	Yes	Yes	Yes
4	Yes	Yes	Yes
5	Yes	No	n/a
6	Yes	No	Yes

*EVG* elastin Von Gieson, *n/a* insufficient sample

There were two notable findings in the study cohort. Firstly, eye number 4 showed a localised area of Descemet’s membrane with Hassall-Henle excrescences associated with the adjacent TM (Fig. 1 c). At the time of surgery incorporation of corneal tissue was not noted and is unavoidable in some instances due to variation in its posterior extension. At follow up no corneal complications were seen. Secondly, eye number 6 with PDG showed dense pigment in every trabecular meshwork cell demonstrating their phagocytic capacity (Fig. 1d). Many pigment granules were also seen on the trabecular beams. All other TM specimens showed much less pigment in TM cells, similar to non-glaucoma eyes.

Two specimens from POAG eyes showed evidence of elastin with EVG staining (Fig. 1e). The remaining three TM specimens did not express elastin. Collagen IV labelling was evident on the surface of trabecular beams in three specimens, identifying the basement membrane underlying the trabecular cells (Fig. 1 f). The other two trabecular specimens were insufficient in size to perform labelling.

## Discussion

TrabEx + can harvest TM with preservation of its architecture. This was demonstrated by the presence of trabecular beams orientated in a lamellar fashion on histology without compression or distortion. Dysfunction of the trabecular meshwork remains only partly understood despite its major pathogenic role in open angle glaucoma. Key observations have included accumulation of extracellular matrix; TM cell loss; changes in many glycosaminoglycan and protein profiles; and increased TM rigidity[5–9]. TrabEx + can harvest TM specimens for applications in immunohistochemistry, proteomics, mass spectrometry, genetic analyses and cell cultures. Tissue studies will direct advances in therapy, and potentially add to the repertoire of medications which will modulate TM structure and function, with Rho kinase inhibitors being the first and only drug class to accomplish this at present[12]. The preservation of tissue integrity in the sampling of trabecular meshwork is a pre-requisite to in-vitro studies and TrabEx + is a suitable non-destructive means of harvesting tissue.

Considering the small sample size and absent control group, deriving conclusions about glaucomatous features on histopathology would be of limited use in this study. Controls derived from non-glaucomatous donor eyes with larger sample numbers would allow meaningful differences to be investigated including variation between glaucoma severities. There are some histopathological findings to note. The absence of elastin in three TM specimens may be due to localisation of elastin in corneoscleral TM which was not included in the specimen. The small nature of the samples can limit investigations, as illustrated with Collagen IV labelling.

.

This study did not specifically address yield of TM retrieval. Nevertheless, it is important to note that tissue retrieval had to be abandoned in some cases of TrabEx+. This often is due to intra-operative anterior chamber haemorrhage, an expected consequence of surgery which obscures angle view. IA maintains good gonioscopic view whilst the TM is stripped. This will facilitate greater degrees of TM removal and minimise inadvertent sample disruption. View thereafter, similar to KDB and TrabEx, is maintained with viscoelastic during tissue retrieval with forceps. During this process the viscoelastic can escape the eye. In such circumstances, the anterior chamber will depressurise and refill with blood. AC washout and more viscoelastic injection are necessary and may need repetition. This will prolong the operation compromising the patient’s tolerance thus necessitating abandoning tissue sampling. Using the aspiration port of the TrabEx + to harvest tissue is a potential method to avoid these challenges. Thereafter, centrifugation of the irrigation fluid could concentrate the sample for histology. Although all material aspirated including blood and other debris would be condensed together which may obscure and distort the fragile TM architecture. This has not been attempted yet. In both methods of tissue harvesting, addressing the percentage yield would need to be addressed in future studies.

TrabEx + reduced both IOP and number of drop treatments, but the change was not statistically significant (*P* > 0.05) for either. The cohort size was poorly powered to measure these changes. However, this was not the main objective of this study and clinical outcomes will be published in future work. It is worth mentioning the poor response of eye number 1. The patient was noted to have dysmorphic features and anomalous anterior segment structures. It is likely that a significant proportion of outflow resistance was distal to the TM, and the eye’s normal TM histology would support this argument. The poor outcome for eye 1 skews the overall IOP outcomes of this small cohort giving large IOP intervals; however the overall IOP outcome are still promising with an average 7.33mmHg reduction in IOP.

The small sample size, absence of control group, and absence of blinding were limitations of this study. Future large cohort studies would allow for correlation between clinical data (i.e. severity, drug therapy, prior laser treatment) and histopathological findings. A natural consequence of TrabEx + derived specimens is the loss of adjacent supporting tissue which could limit studies on TM function. The risk of tissue distortion or destruction, not demonstrated in this study, still remains a potential risk to larger cohort studies. The small and fragile nature of the specimens may be insufficient to perform particular laboratory studies and the in vitro behaviour of TM cells does not always replicate in vivo behaviour[13].

## Conclusions

TrabEx + is a viable means to harvest trabecular meshwork with tissue preservation allowing histopathological and further laboratory studies.

## Data Availability

The datasets used and analysed during the current study are available from the corresponding author on reasonable request.

## Abbreviations

USA: United States of America
TM: Trabecular meshwork
IA: Irrigation and aspiration
KDB: Kahook Dual Blade
POAG: Primary open angle glaucoma
SC: Shlemm’s canal
OVD: Ophthalmic viscosurgical device
NSOPS: National Specialist Ophthalmic Pathology Service (NSOPS)
OHT: Ocular hypertension
UK: United Kingdom
IOP: Intraocular Pressure
AC: Anterior chamber
H&E: Haematoxylin and eosin
PAS: Periodic acid-Schiff
EVG: Elastin Von Gieson
IHC: Immunohistochemistry
TRS: Target Retrieval Solution
PDS: Pigment dispersion glaucoma
